# In Vitro Screening for Probiotic Properties of *Lactobacillus* and *Bifidobacterium* Strains in Assays Relevant for Non-Alcoholic Fatty Liver Disease Prevention

**DOI:** 10.3390/nu15102361

**Published:** 2023-05-18

**Authors:** Silvia Lopez-Escalera, Mari L. Lund, Gerben D. A. Hermes, Béatrice S.-Y. Choi, Kei Sakamoto, Anja Wellejus

**Affiliations:** 1 Human Health Research, Scientific Affairs, Chr. Hansen A/S, Bøge Alle 10-12, 2970 Hørsholm, Denmark; 2 Fakultät für Biowissenschaften, Friedrich-Schiller Universität Jena, Bachstraβe 18K, 07743 Jena, Germany; 3 Department of Biomedical Sciences, Faculty of Health and Medical Sciences, University of Copenhagen, 2200 Copenhagen, Denmark; 4 Novo Nordisk Foundation Center for Basic Metabolic Research, University of Copenhagen, 2200 Copenhagen, Denmark

**Keywords:** probiotics, lactobacillus, bifidobacterium, NAFLD, gut health, GLP-1, intestinal organoids, microbial metabolomics, bacteria–host interaction, de novo lipogenesis

## Abstract

Non-alcoholic fatty liver disease (NAFLD) is a multifactorial metabolic disorder that poses health challenges worldwide and is expected to continue to rise dramatically. NAFLD is associated with metabolic syndrome, type 2 diabetes mellitus, and impaired gut health. Increased gut permeability, caused by disturbance of tight junction proteins, allows passage of damaging microbial components that, upon reaching the liver, have been proposed to trigger the release of inflammatory cytokines and generate cellular stress. A growing body of research has suggested the utilization of targeted probiotic supplements as a preventive therapy to improve gut barrier function and tight junctions. Furthermore, specific microbial interactions and metabolites induce the secretion of hormones such as GLP-1, resulting in beneficial effects on liver health. To increase the likelihood of finding beneficial probiotic strains, we set up a novel screening platform consisting of multiple in vitro and ex vivo assays for the screening of 42 bacterial strains. Analysis of transepithelial electrical resistance response via co-incubation of the 42 bacterial strains with human colonic cells (Caco-2) revealed improved barrier integrity. Then, strain-individual metabolome profiling was performed revealing species-specific clusters. GLP-1 secretion assay with intestinal secretin tumor cell line (STC-1) found at least seven of the strains tested capable of enhancing GLP-1 secretion in vitro. Gene expression profiling in human biopsy-derived intestinal organoids was performed using next generation sequencing transcriptomics post bacterial co-incubation. Here, different degrees of immunomodulation by the increase in certain cytokine and chemokine transcripts were found. Treatment of mouse primary hepatocytes with selected highly produced bacterial metabolites revealed that indole metabolites robustly inhibited de novo lipogenesis. Collectively, through our comprehensive bacterial screening pipeline, not previously ascribed strains from both Lactobacillus and Bifidobacterium genera were proposed as potential probiotics based on their ability to increase epithelial barrier integrity and immunity, promote GLP-1 secretion, and produce metabolites relevant to liver health.

## 1. Introduction

The current worldwide prevalence and expected growth of chronic liver disease incidence poses a major health risk and economic burden on society, calling for urgent multidisciplinary approaches to manage further increase. Therefore, there is an urgent need for novel approaches to manage this complex disease [1]. Non-alcoholic fatty liver disease (NAFLD), recently renamed as metabolic-associated fatty liver disease (MAFLD), ref. [2] has become the most common hepatic disease, currently affecting 25% of the global population and is forecasted to expand in parallel with cardiometabolic diseases such as obesity and type 2 diabetes mellitus (T2DM) [3,4]. NAFLD includes a spectrum of liver abnormalities covering simple hepatosteatosis and steatohepatitis with fibrosis involvement, known as non-alcoholic steatohepatitis (NASH). NAFLD has been associated with various metabolic dysfunctions ranging from alterations in glucose-, lipid- and bile acid metabolism, hepatic inflammation, insulin resistance, gut microbiota composition, and intestinal permeability (i.e., leaky gut) [5,6,7,8]. Numerous studies have evaluated the interplay between gut microbes and development of host-metabolic diseases, showing possible associations between commensal bacteria and disease development [9,10,11,12]. Due to the intensive search for effective medical treatment, NAFLD is currently extensively studied [1,4,13].

The essential crosstalk between the gut and liver regulates inter-organ homeostasis, which is important for host well-being [14]. In healthy subjects, an intact intestinal epithelial lining serves as a protective barrier against translocation of bacteria, bacterial endotoxins (lipopolysaccharides), and pathogen-associated molecular patterns, to peripheral organs. Conversely, a disrupted gut barrier can result in the transport of these potentially harmful components [15,16] to the liver via the portal vein, which can trigger downstream cascades of immunological reactions which induce steatosis, and eventually fibrosis formation, leading to liver injury [14,17,18]. Bacterial metabolites, both present in the intestine and those that are transported to the liver, are of great importance for human health and have been employed as biomarkers for disease development [19,20]. For example, short-chain fatty acids (SCFAs) and indoles are well-studied microbial-derived by-products that exert beneficial effects on the host [21,22]. Acetate, propionate, and butyrate are some of the main SCFAs produced by the gut microbiome which result from the fermentation of non-digestible dietary fibers, whereas indoles are generated by microbial catabolism of tryptophan [23,24]. Decreased levels of SCFAs have been associated with development of NAFLD by inducing hepatic fatty acid synthesis and promoting gluconeogenesis and inflammation [25,26,27]. Indoles act as signaling molecules, for example, through the activation of aryl hydrocarbon receptor (AhR), and emerging evidence indicates that indole-3-acetic acid alleviates high-fat diet-induced NAFLD in mice [28]. In addition, it has been observed that a reduction in intestinal AhR activation is associated with the development of metabolic diseases, further supporting the critical role of indoles for healthy systemic homeostasis [19,29]. Both SCFAs and indoles have shown the potential to modulate the secretion of gut hormones, particularly glucagon-like peptide-1 (GLP-1), albeit through different mechanisms [30,31,32]. GLP-1 exerts pleiotropic effects by controlling glucose-dependent insulin secretion, β-cell mass, satiety sensation, and gastric emptying [33,34], and molecules triggering the release of GLP-1 are applicable for management of a wide range of metabolic conditions [31,35,36]. Multiple preclinical and clinical studies with GLP-1 agonist treatment resulted in beneficial effects on hepatic inflammation, steatosis, and fibrosis [37], thus, an increase in circulating GLP-1 could be a therapeutical advantage.

Treatment of NAFLD is typically focused on managing related conditions such as obesity, hyperlipidemia and T2DM by modifying lifestyle habits such as physical activity and diet [10,38,39]. However, given the diverse functional features of the gut microbiota and the growing body of evidence linking the gut microbiome and NAFLD, probiotic supplementation may serve as an alternative therapeutic or preventative option, by modulating intestinal epithelial integrity, gut hormone release or metabolite profiles [22,40,41]. Probiotics are defined as “live microorganisms that, when administered in adequate amounts, confer a health benefit on the host” [42], and the most commonly used probiotics belong to the genera Bifidobacterium and Lactobacillus [36,43]. Safe use of probiotics has been documented for decades, addressing a wide range of conditions, such as irritable bowel syndrome, inflammatory bowel disease, antibiotic-associated diarrhea, and respiratory tract infections [44,45,46,47]. In addition, multiple probiotic strains have been tested for modulation of NAFLD and T2DM with varying outcomes, and although recent meta-analyses suggest that probiotics can reduce HbA1c, fasting blood glucose and insulin resistance in T2DM patients [48] and reduce aminotransferases in NAFLD patients [49], efficacy seems to be strain dependent, suggesting that a careful preclinical screening of potential metabolically relevant targets would increase the likelihood of identifying a beneficial bacterial strain.

Therefore, we screened a collection of novel bacterial strains as well as already recognized probiotic strains in multiple in vitro setups, all physiologically relevant with respect to increasing liver health. This novel screening pipeline will aid the selection of clinically relevant strains by evaluating metabolome, impact on intestinal barrier integrity, induction of GLP-1 secretion, and gene expression in human biopsy-derived organoids. Subsequently, selected bacterial metabolites were assessed for inhibition of hepatic lipogenesis. Via this bacterial screening approach, novel bacterial candidates that may promote liver health in pathological conditions such as NAFLD, were identified.

## 2. Materials and Methods

### 2.1. Cell Culture

Human colon adenocarcinoma Caco-2 cell line (DSMZ ACC 169) was cultured in Dulbecco′s Modified Eagle′s Medium (DMEM, Gibco™) supplemented with 20% (*v*/*v*) Fetal Bovine Serum (FBS, Gibco™), 1% (*v*/*v*) MEM non-essential amino acids (Biowest), and 1% (*v*/*v*) Pen-Strep-Amp B (AB, Biological Industries, Cromwell, CT, USA), hereafter referred to as complete DMEM. Cells were incubated at 37 °C in humidified conditions with 5% CO_2_, the medium was renewed biweekly, and cells were subcultivated upon reaching 80% confluency. Cell passage numbers ranging between 6 and 30 were used for these assays.

The heterogenous plurihormonal cell line STC-1 (intestinal secretin tumor, CRL-3254 ™) was cultured with Dulbecco′s Modified Eagle′s Medium (DMEM) supplemented with 10% (*v*/*v*) Fetal Bovine Serum (FBS, Gibco™), and 1% AB (*v*/*v*). Cells were grown and maintained at 5% CO_2_ at 37 °C with medium renewal every 2 to 3 days. Cells were passaged when cell confluency reached 70%, and the passage number used for this assay was between 6 and 34. All mammalian cell work was conducted using sterile technique on LAF benches.

### 2.2. Bacterial Strains and Culture

Based on previously reported beneficial results in NAFLD clinical and animal studies, specific bacterial species from the Lactobacillus and Bifidobacterium genera were selected, and from these species, 42 strains from the Chr. Hansen culture collection were included in this study (Supplementary Table S1). All strains were inoculated from frozen stock in De Man, Rogosa and Sharpe broth (MRS), pH 6.5 (Difco™), and cultured overnight at 37 °C. For bifidobacteria, MRS was supplemented with 0.05% Cysteine hydrochloride monohydrate (CyHCl) and incubated under anaerobic conditions. Overnight cultures were subcultured using 10-fold dilutions and incubated at 37 °C overnight. For each strain, samples of late exponential/early stationary phase (determined by OD measurements of ON dilution row cultures) were pooled and centrifuged at 4500 rpm for 10 min at 20 °C to collect the bacterial pellet. Pellets were washed with PBS twice before final resuspension in warm complete DMEM AB-free, and the final OD600 was adjusted to 4. To measure colony forming units (CFU) in the OD-adjusted bacterial solutions, one mL of each culture was diluted in Peptone water (maximum recovery diluent cups, MRD; Oxoid) and a 10-fold dilution series was prepared and deep-seeded in MRS agar plates. The media were supplemented similarly as previously described according to genus. Plates were incubated at 37 °C for 2 days and colonies were counted and CFUs were calculated. Technical duplicates were prepared of each dilution. All bacterial work was conducted under well-established hygiene conditions to avoid potential cross-contamination of strains. All strains were inoculated from cryostocks originally made from single-picked colonies. Upon CFU testing, colonies were visually inspected for possible contaminants and control media was additionally plated to verify that no unspecific growth was observed.

### 2.3. Transepithelial Electrical Resistance (TEER)

Prior to the transepithelial electrical resistance (TEER) experiments, the apical compartments of a 12-well, 12 mm transwell with a 0.4 µm pore polyester membrane insert (Corning, Corning, NY, USA) were seeded with 105 Caco-2 cells. These cells were kept under the same conditions as mentioned earlier and maintained with 500 µL apical and 1500 µL basolateral culture medium for 21 days with medium renewal twice per week. The day before TEER measurements, cultured transwells were transferred to the CellZscope2 (Nanoanalytics Germany, Münster, Germany) and the cell culture media was fully replaced by adding 800 µL apical and 1500 µL basolateral fresh complete DMEM AB-free. The CellZscope2 was kept overnight in a humidified atmosphere at 37 °C with 5% CO_2_.

Caco-2 cells were co-incubated with single Lactobacillus and Bifidobacterium strain-solutions to explore the impact on the TEER. Transwells were transferred to the CellZscope2 the day before bacterial exposure to establish baseline TEER readings for each well, which also served as quality control of a stable electrical resistance. On the day of the experiment, the CellZscope2 was paused and 100 µL of apical medium was replaced with either 100 µL of bacterial cell suspension (final OD600 = 0.5) or complete DMEM AB-free in triplicates, respectively. The CellZscope2 was kept in a humidified atmosphere at 37 °C with 5% CO_2_, and TEER readings were resumed with continuous hourly readings for a total of 18 h. Apical supernatants were collected by the end of the TEER experiments and stored at −80 °C for further analysis.

Additionally, individual bacterial metabolites (details in Supplementary Table S2) were tested in the TEER after dissolving the metabolites in the control medium, complete DMEM AB-free alone. All concentrations were tested in triplicate. The cell-free supernatants tested were spent medium from ON anaerobic incubation, centrifuged and sterile filtered before being added to the CellZscope2 in the same volume as live bacteria.

### 2.4. Metabolic Profiling of the Probiotic Strains

To study which metabolites impacted the TEER increase, we measured metabolites of the apical supernatants after the Caco-2 co-incubation with bacteria. Short chain fatty acids (SCFAs) and semi-polar metabolites were measured (MS-Omics, Hørsholm, Denmark). For SCFA analysis, samples were acidified using hydrochloride acid, and deuterium labelled internal standards were added. All samples were analyzed in a randomized order. Analysis was performed using a high polarity column (Zebron™ ZB-FFAP, GC Cap. Column 30 m × 0.25 mm × 0.25 µm) installed in a GC (7890B, Agilent) coupled with a quadrupole detector (5977B, Agilent). The system was controlled by ChemStation (Agilent, Santa Clara, CA, USA). Raw data were converted to netCDF format using Chemstation (Agilent), before the data were imported and processed in Matlab R2014b (Mathworks, Inc., Natick, MA, USA) using the PARADISe software described by [50].

Semi-polar metabolites analysis was carried out using a UPLC system (Vanquish, Thermo Fisher Scientific, Waltham, MA, USA) coupled with a high-resolution quadruple-orbitrap mass spectrometer (Q Exactive™ HF hybrid quadrupole-orbitrap, Thermo Fisher Scientific). An electrospray ionization interface was used as an ionization source. Analysis was performed in negative and positive ionization mode. The UPLC was performed using a slightly modified version of the protocol previously described [51]. Peak areas were extracted using Compound Discoverer 3.1 (Thermo Scientific, Waltham, MA, USA). The identification of compounds was performed at four levels, namely, level 1: identification by retention times (compared against in-house authentic standards), accurate mass (with an accepted deviation of 3 ppm), and MS/MS spectra; level 2a: identification by retention times (compared against in-house authentic standards), accurate mass (with an accepted deviation of 3 ppm); level 2b: identification by accurate mass (with an accepted deviation of 3 ppm), and MS/MS spectra; level 3: identification by accurate mass alone (with an accepted deviation of 3 ppm). Annotations for level 3 were based on searches in the Yeast Metabolome Database.

Machine learning analyses were performed in R version 4.2.0 [52]. To determine which metabolites were strongly associated with a normalized TEER after 8 h, we used random forest (RF) regression based on metabolite peak areas. We evaluated the model using repeated cross-validation as implemented in the caret package [53]. We randomly selected 20% of the samples to serve as the training set and consequently evaluated model accuracy using the hold out set. This was repeated 5 times. The optimal model was selected, and the top 35 most important predictors based on mean decrease accuracy (%IncMSE) were plotted. %IncMSE depicts how much the model accuracy decreases if that variable is left out.

### 2.5. GLP-1 Total Secretion Studies

Cultured STC-1 cells were rinsed with sterile-filtered HEPES buffer (140 mM NaCl, 4.5 mM KCl, 1.2 mM CaCl_2_, 1.2 mM MgCl_2_, 20 mM HEPES) and dissociated with 2 mL Trypsin EDTA (TrypLE Express Enzyme; Gibco™) for 10 min in a humidifier incubator. Cells were resuspended in 6 mL growth medium and the cell pellet was spun down at 125× *g* for 5 min. The supernatant was discarded and the pellet was resuspended in fresh growth medium. STC-1 cells were seeded at 5 × 105 cells/well on 12-well plates (Costar, Washington, DC, USA) and the cultures were incubated overnight at 37° at 5% CO_2_. STC-1 cells were co-incubated with each of the 42 bacterial strains, grown under similar conditions as described previously with a final OD600 adjustment of 7.5 in HEPES buffer. Prior to treatment, all STC-1 cell layers were rinsed with 1 mL/well HEPES buffer. For the co-incubation assay, a final volume of 1.5 mL/well was desired. Cells were incubated with 1.380 mL/well HEPES buffer plus 100 µL bacteria suspension (final OD600 = 0.5) for 3 h in a humidified atmosphere as described above. A positive control solution was prepared according to [54], containing glutamine 40 mM, valine 40 mM, lysine 40 mM, glucose 40 mM, and fructose 40 mM in HEPES buffer. Moreover, 10 µL DPP IV (Sigma, St. Louis, MO, USA; DPP4-010) was added before and after incubation in each well.

After incubation, 150 µL supernatant from each well was transferred to an ice-cold 96-well plate and spun down for 5 min, at 4 °C and 1500 rpm. Cell-free supernatants were transferred to a fresh ice-cold 96-well-plate and stored at −80 °C until analysis. A U-PLEX mouse GLP-1 (total) assay (Meso Scale Diagnostics, LLC, Rockville, MD, USA; K1525UK-2) was performed according to the manufacturer’s protocol. The U-PLEX plates were read using an MSD instrument (Meso QuickPlex SQ 120) and the data were analyzed using software MSD Discover Workbench 4.0.12 (LSR_4_0_12). Treatments were made in triplicate and means were normalized to the negative control = 1 (HEPES buffer).

### 2.6. Human Small Intestinal Organoid Studies

#### 2.6.1. Human Small Intestinal Organoid Culture

Human small intestinal organoids (HIO) were derived from healthy subjects’ biopsies under previous work funded by the European Union’s Horizon 2020 research and innovation program (STEMHEALTH ERCCoG682665). In this study, HIO were thawed and cultivated as 3D spheres (domes) by embedding the organoid culture solution in Matrigel (Corning; 356231) at a 1:1 ratio in a 24-well plate (Costar; 3526). HIO cultures were maintained in 750 µL Human IntestiCult™ Organoid Growth Medium (StemCell Technologies, Vancouver, BC, Canada; 06010) with 100 U-mg/mL penicillin–streptomycin (P/S, Sigma; P4333), hereafter referred to as OGM complete, incubated at 37 °C, 5% CO_2_. The medium was replaced every second day and subcultivation was performed after 7–10 days in culture or whenever more cells were needed, until numerous buds were observed per dome. According to StemCell Technologies protocol, subculturing was performed by dissociating organoid domes. The medium was removed and 500 µL Gentle Cell Dissociation Reagent (Stemcell Technologies; 07174) was added to each well and incubated for 1 min at RT. Organoid dome disruption was performed mechanically by gentle pipetting and then was transferred to a conical Greiner CellStar tube pre-coated with 1% (*v*/*v*) BSA (Sigma) in DMEM F-12 (Stemcell Technologies). For organoid expansion, domes yielding 150–200 mature organoids were harvested and up to 4 domes were pooled. Organoid suspension was kept on a rocking platform for 10 min at RT on gentle motion (20 rpm) and centrifuged at 290× *g* for 5 min at 4 °C. The supernatant was discarded and fresh ice-cold 1% BSA DMEM F-12 was added and spun down as previously described. The pellet was resuspended, and the organoid suspensions were passed through a 70 µm cell strainer (StemCell; 27216) to obtain a uniform cell suspension. The cell suspensions were centrifuged at 200× *g* at 4 °C for 5 min, the media were removed, and pellets were resuspended in 50 µL/well OGM complete medium supplemented with 10 µM Y-27632 dihydrochloride (Sigma) followed by the addition of 50 µL/well ice-cold Matrigel. New organoid spheres were prepared by carefully plating 50 µL of single cell matrix per well. Cultured plates were incubated for 10–15 min, allowing for the Matrigel to solidify, followed by adding 750 µL OGM complete medium with Y-27632.

#### 2.6.2. Organoid Monolayer

For the establishment of HIO 2D monolayers, 48-well plates (Costar) were coated with a solution of Matrigel (diluted 1:50 in ice-cold PBS) and incubated for 1 h at 37 °C before use. Single cell suspensions were prepared as described previously with a few modifications. Organoid fragments were enzymatically dissociated by resuspending the pellet in 5 mL of 37 °C 0.05% Trypsin-EDTA (Stemcell Technologies; 07910). Dissociation to single cell suspensions was confirmed via inverted microscopy. An equal amount of DMEM F-12 was added and mixed thoroughly, followed by centrifugation 300× *g* at 4 °C for 5 min. To induce cell differentiation, pelleted single cells were resuspended in Intesticult™ Organoid Differentiation Medium (ODM, Stemcell Technologies) supplemented with Y-27632 plus P/S. Before plating single cells, excess Matrigel solution was aspirated, and cell suspension was seeded on a 48-well Matrigel coated plate. The cultures were incubated at 37 °C, 5% CO_2_ until the 2D cell monolayers reached confluency with ODM replacement every second day. Organoid passage number ranged from 11 to 16.

#### 2.6.3. Organoid–Bacterial Co-Culture

Bacterial strains were cultured as previously described. PBS-washed bacterial pellets were resuspended in ODM with Y-27632 without P/S, and OD600 was adjusted to 1. Two dimensional monolayers were washed once with 300 µL DMEM F-12 and co-incubated with 300 µL of bacterial suspension for 2 h under previously described conditions.

#### 2.6.4. RNA Extraction from Organoids

HIO RNA isolation was performed by applying 500 µL TRIzol™ reagent (Invitrogen, Washington, DC, USA; 15596026) to each well and disrupting the monolayer mechanically by pipetting. Monolayers were incubated for 5 min at RT and transferred to Eppendorf tubes. An amount of 100 µL chloroform was added and the tubes were shaken for 15 s and placed at RT for 3 min. The cell lysates were centrifuged at 12,000× *g* for 15 min at 4 °C and the upper aqueous phases were transferred to new RNase-free 1.5 mL Eppendorf tubes. An amount of 1 µL of 20 mg/mL Rnase-free glycogen (Invitrogen) and 250 µL isopropanol (Sigma) were added, and tubes were mixed vigorously for 15 s. The homogenous solutions were incubated for 10 min at RT and were centrifuged at the previously described settings. Supernatants were discarded and RNA pellets were washed in 500 µL 75% ethanol 3 times with tube inversion, and subsequently centrifuged for 5 min, 7500× *g* at 4 °C. After removing the supernatant, RNA pellets were air dried for 5–10 min and eluted in 20 µL Rnase-free water. RNA was quantified using the NanoDrop One (Thermo Scientific). Pellets were purified with Dnase according to the manufacturer’s recommendations (Qiagen, Hilden, Germany, 1023460), eluted in 15 µL Rnase-free water, and stored at −80 °C.

#### 2.6.5. RNA-Seq-Transcriptomics

RNA was sent to AZENTA Life Sciences (Leipzing, Germany) for whole transcriptomic analysis (RNA-seq). Sequencing libraries were prepared by the removal of rRNA via PolyA selection and subsequently sequenced on an Illumina NovaSeq. Sequence reads were trimmed using Trimmomatic v.0.36 and trimmed reads were mapped to the Homo sapiens GRCh38 reference genome available on ENSEMBL using the STAR aligner v.2.5.2b. Unique gene hit counts were calculated by using featureCounts from the Subread package v.1.5.2. Using DESeq2, a comparison of gene expression between the control and treatment groups of samples was performed. The Wald test was used to generate *p* values on log2 transformed data. Genes were considered differentially expressed with an adjusted *p*-value < 0.1 and log2 fold change >1 for each comparison. Gene ontology (GO) was used for pathway analysis of gene expression.

### 2.7. Mouse Primary Hepatocyte Isolation, Culture and Lipogenesis Assay

Animal studies were performed in accordance with the European directive 2010/63/EU of the European Parliament and the Council of the Protection of Animals used for scientific research. The Danish Animal Experiment Inspectorate provided ethical approval (#2021-15-0201-00884). Primary hepatocytes were obtained from 8 week old male C57BL/6 NTac mice, and the isolation method used was described previously [55,56]. The cells were seeded at a density of 5 × 10^5^ cells/well on collagen-coated 6-well plates (Corning; 354400) and kept in the same humified conditions for 4 h to allow the hepatocytes to attach. After attachment, the medium was replaced by M199 supplemented with 1% Penicillin/Streptomycin, 1 µM dexamethasone (Sigma-Aldrich; D4902), and 1 nM insulin (Sigma-Aldrich, I3278) referred to as culture medium, for overnight cell culture at 37 °C with 5% CO_2_. Cultured hepatocytes were treated with indole metabolites: indole-3 lactic acid (ILA, Sigma; I5508), indole-3 carbaldehyde (ICA, Sigma; I29445), and indole-3 acetaldehyde (IAL, Sigma; I1000), at 0.5 mM, 1 mM and 5 mM, respectively. An allosteric activator of AMPK (MK-8722) [57] was used as a positive control, and vehicle-treated cells were used as negative control. All treatments were diluted in cultured medium and incubated at 37 °C with 5% CO_2_ for 3 h. De novo lipogenesis was assessed in primary hepatocytes according to [56] method by adding a final concentration of 0.12 μCi of [^14^C]-acetate tracer directly in the medium of all treatments. [^14^C]-acetate was measured after reconstitution of the lipid extracts in Ultima gold scintillation fluid. Data reflect the incorporation of [^14^C]-acetate into lipids, mainly fatty acids. Results were normalized to total protein content.

### 2.8. Statistical Analysis

Results are presented as the mean of triplicates with standard deviation (SD) as error bars. In the TEER co-incubation experiments, the area under the curve (AUC) for all the strains at t = 8 h were assessed and compared with the unstimulated control via a one-way ANOVA with Dunnett’s post-hoc analysis for multiple comparisons. Data for GLP-1 release and lipogenesis were analyzed using a one-way ANOVA followed by Dunnett’s post-hoc analysis for multiple comparisons. Statistical significances were considered at *p* values < 0.05. Data analysis and visualization were performed using the software GraphPad Prism (v. 9.3.1 GraphPad Software, LLC.) and R (version 4.2.0), respectively.

## 3. Results

### 3.1. Transepithelial Electrical Resistance Response (TEER) to Bacterial Co-Incubation

A total of 42 *Lactobacillus* or *Bifidobacterium* strains, from the Chr. Hansen A/S culture collection, were selected and included in this screening study (Supplementary Table S1). The selection was partly based on previously reported bacterial species with beneficial results in NAFLD clinical and animal studies, and partly based on finding novel candidate probiotic strains.

**Figure 1:**
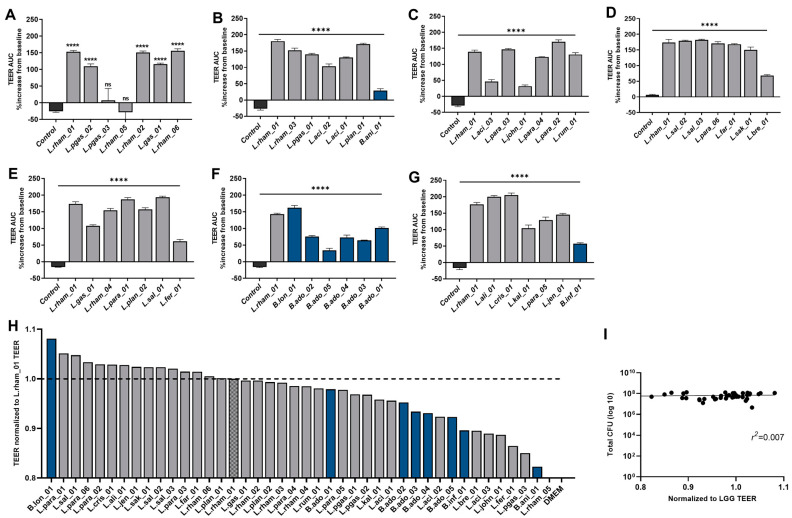
Modulation of barrier integrity by 42 individual bacterial strains. (**A**–**G**) Results from individual TEER runs shown as TEER area under the curve after 8 h normalized to percentage increase from baseline measurements. Blue bars correspond to *Bifidobacterium* strains, and dark gray to *Lactobacillus* strains. Co-cultures were tested in triplicate and error bars indicate SD. Results were compared with the unstimulated control via a one-way ANOVA with Dunnett’s post-hoc analysis for multiple comparisons: **** *p* < 0.0001, ns: not significant. (**H**) The 42 bacterial strains ranked according to respective TEER readings of Caco-2 monolayers at 8 h incubated with viable bacteria. Data were normalized to positive control (L.rham_01 = 1; dotted line). Each blue bar corresponds to *Bifidobacterium* strains, and dark gray to *Lactobacillus* strains. DMEM AB-free media was used as a negative control. Bars represent the mean of triplicates. (**I**) Association between added colony forming units (CFU) and TEER response tested by linear regression.

Through co-incubation of mature colonic Caco-2 cell monolayers with 42 viable bacterial strains, individually, barrier integrity was evaluated by real-time measurements of TEER values over a period of 18 h (Supplementary Figure S2). Prior to bacterial co-incubation, the TEER stabilized for 24 h to values ranging between 300 and 450 Ω × cm^2^. Changes in TEER during bacterial stimulation were calculated relative to baseline value (=100%) recorded immediately before the addition of bacteria. The strain named LGG^®^, *Lactocaseibacillus rhamnosus* (L.rham_01) is a well-documented probiotic strain reported to support intestinal barrier function [58], and, therefore, this strain was included as a positive control in all runs of the TEER screening. The mean TEER area under the curve (AUC) after 8 h for each strain was compared with Caco-2 baseline levels and all strains, except two (L.rham_05 and L.pgas_03), significantly increased TEER AUC (Figure 1A–G).

The bacterial strains were ranked according to their epithelial strengthening capacity after 8 h of co-incubation with Caco-2 cells (Figure 1H). Here, TEER AUC 8 h after stimulation with bacterial strains were normalized to the mean TEER AUC for L.rham_01 (L.rham_01 = 1) for each experiment. A total of 15 bacterial strains enhanced barrier integrity above the level of L.rham_01, and of these, 14 strains were lactobacilli, such as L.para_01 (ratio = 1.05) and L.sal_01 (ratio = 1.04). Only one *Bifidobacterium* strain, B.lon_01, ranked higher than L.rham_01, and surprisingly it showed the strongest TEER increase (ratio = 1.081) of all the strains, whereas the rest of the tested *Bifidobacterium* strains ranked below the positive control. None of the 42 strains in the screening provoked damage to the Caco-2 monolayers under the tested conditions.

Moreover, colony forming units (CFUs) were determined in the bacterial suspensions added to the TEER (Figure 1I). No association was found (*r*^2^ = 0.007) between the number of live bacteria added and the TEER response.

### 3.2. Metabolite Profiles of Screened Bacteria Strains

**Figure 2:**
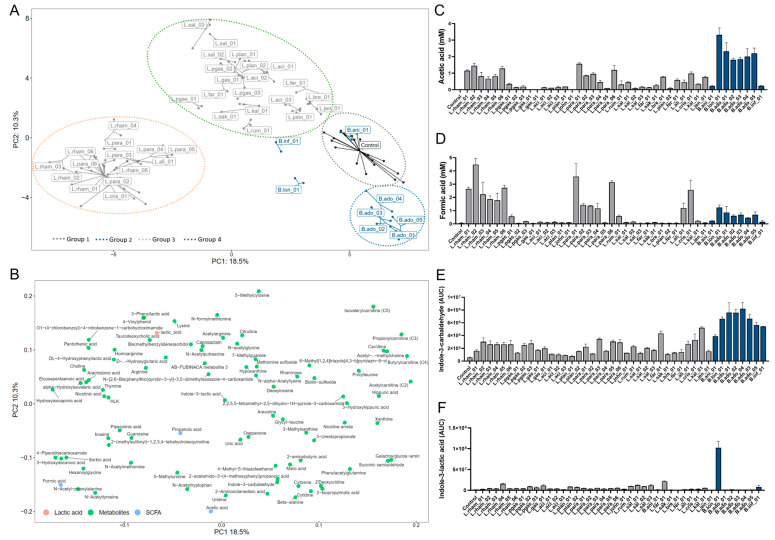
Bacterial-derived metabolite profiles. (**A**) Principal component analysis (PCA) of metabolites (level 1, 2a, 2b and SCFAs) labeled by strain ID and colored by genus: gray = *Lactobacillus* strains, blue = *Bifidobacterium* strains, black = controls (*n* = 21). (**B**) Metabolite loading plot showing the metabolites driving sample-position on PC1 and PC2 (blue = SCFAs, green = semi-polar metabolites). (**C**,**D**) Quantified acetic acid and formic acid production post co-incubation. Blue bars correspond to *Bifidobacterium* strains, and dark gray to *Lactobacillus* strains. All strains were tested in triplicate and error bars indicate SD. (**E**,**F**) Production of the microbial-derived tryptophan catabolites: Indole-3-carbaldehyde (ICA) and Indole-3-lactic acid (ILA) depicted as AUC. Blue bars correspond to *Bifidobacterium* strains, and dark gray to *Lactobacillus* strains. All strains were tested in triplicate and error bars indicate SD.

In order to evaluate the microbial- and Caco-2-derived metabolites produced in the TEER assay, supernatants from the apical side of the transwells were analyzed. Untargeted, semi-polar metabolite analysis detected a total of 764 compounds. Of these, 48 were annotated on level 1, 64 on level 2a, 54 on level 2b and 24 on level 3. The remaining were unidentified. Principal component analysis (PCA) was performed on characterized compound classes (level 1, 2a and 2b) and SCFAs from supernatants of the 42 tested strains and controls. The first two principal components explained 18.5% (PC1) and 10.3% (PC2) of the total variation (Figure 2A,B), respectively. Four groups were observed based on similarities in metabolite profiles based on the loading plot (Figure 2B). Group 1 included the control samples and one *Bifidobacterium* strain, B.ani_01. This strain displayed a metabolite profile similar to the control samples without bacteria. Group 2 contained the six *B. adolecentis* strains. Group 3 consisted primarily of the positive control L.rham_01 (LGG^®^, *Lactocaseibacillus rhamnosus)*, other *L. rhamnosus* strains and *L. paracasei* strains. Group 4 contained the remaining *Lactobacilli* strains. These groups underline the similarities of metabolite profiles at the species level. Additionally, two *Bifidobacterium* strains, B.lon_01 and B.inf_01, clustered separately, indicating that these strains produced distinct metabolic profiles. The main metabolite components driving the segregation of the four major groups found in the PCA plot are described by the loading plot (Figure 2B). For instance, the production of acetic acid (Figure 2C) and Indole-3-carbaldehyde (Figure 2D) were some of the components differentiating Group 2 (*B. adolescentis* strains) from the other groups. Another important metabolite in differentiating the different groups was formic acid, a SCFA which was identified in a genus- and species-specific pattern, and mainly produced by *L. rhamnosus, L. paracasei, L. alimentis* and *L. crispatus* strains (Figure 2D). Conversely, lactic acid was identified in the supernatants of all samples. Tryptophan derivates and other aromatic metabolites were only produced by certain bifidobacteria. High levels of indole-3-carbaldehyde (ICA) were produced by all *Bifidobacterium* species, whereas indole-3-acetaldehyde (IAA) and indole-3-lactic acid (ILA) were solely produced by B.lon_01 (Figure 2F), thus being some of the metabolites driving the separation of *B. longum* from the groups.

### 3.3. Prediction of Microbial-Derived Metabolites Important for Intestinal Barrier Integrity

**Figure 3:**
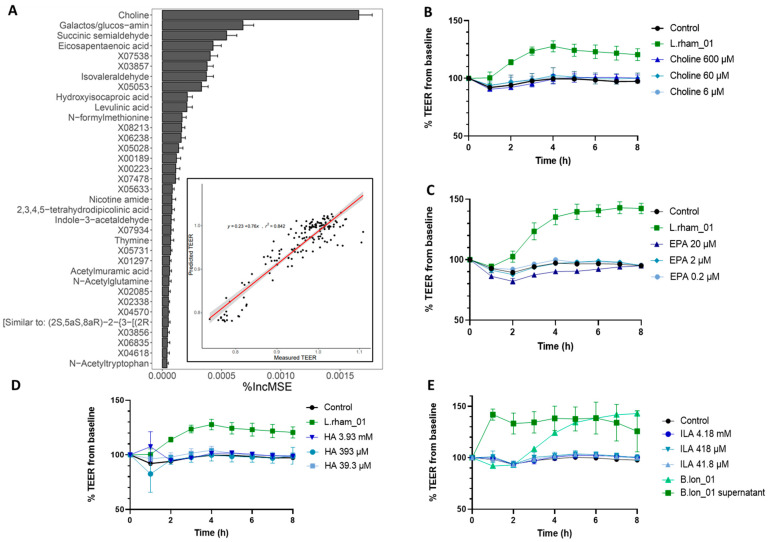
Prediction and testing of metabolites correlating with TEER increase. (**A**) Most important bacterial metabolites for the prediction of normalized TEER after 8 h by random forest regression. %IncMSE represents the relative impact of a metabolite on the accuracy of the model. Model performance shown inside the RF plot. The red line is fitted by linear regression. (**B**–**E**) Transepithelial electrical resistance (TEER) readings of Caco-2 monolayers challenged with single metabolites for 8 h with DMEM AB-free used as control. Error bars indicate the SD of *n* = 3. HA, hydroxysiocaproic acid; EPA, Eicosapentanoeic acid; ILA, Indole-3-lactic acid.

To investigate bacterial metabolites responsible for increases in TEER, a machine learning approach was employed. Random forest (RF) regression was applied to identify metabolites that were strongly associated with the observed TEER. RF can also identify non-linear relationships. The RF model was most predictive using TEER readings after 8 h (*r*^2^ = 0.842) (Figure 3A insert). The top 35 most important predictors based on mean decrease accuracy (%IncMSE) were plotted. %IncMSE is a measure of importance and depicts how much model accuracy decreases if that variable is left out. Choline, galactos/glucose-amin, succinic semialdehyde and eicosapentanoeic acid (EPA) were most predictive for measured TEER (Figure 3A). Choline, EPA and hydroxyisocaproic acid (HA) were subsequently quantified and tested individually in the TEER model in relevant concentrations (Figure 3B–D). Surprisingly, none of the tested metabolites induced a significant TEER response compared with unstimulated Caco-2 monolayers, suggesting that although measured TEER was strongly associated with the concentrations of these metabolites, they were not responsible for the observed effects or that a specific combination of different metabolites is essential.

Since B.lon_01 induced the highest TEER, we analyzed the specific metabolic profile of the strain and found high production of the indole, ILA, a metabolite with known beneficial effects on gut physiology. Therefore, concentrations of ILA were quantified and its effect on TEER was measured. Again, no increase in TEER response over 8 h was observed (Figure 3E). In contrast, viable B.lon_01 and cell-free B.lon_01 supernatant increased TEER significantly compared with untreated controls. Remarkably, the cell-free B.lon_01 supernatant induced maximum TEER readings after only 1 h (141.1 Ω × cm^2^), compared with the viable bacteria that induced max TEER readings after 8 h as previously observed (142 Ω × cm^2^).

### 3.4. Bacterial Stimulation of Glucagon-like Peptide-1 Secretion

**Figure 4:**
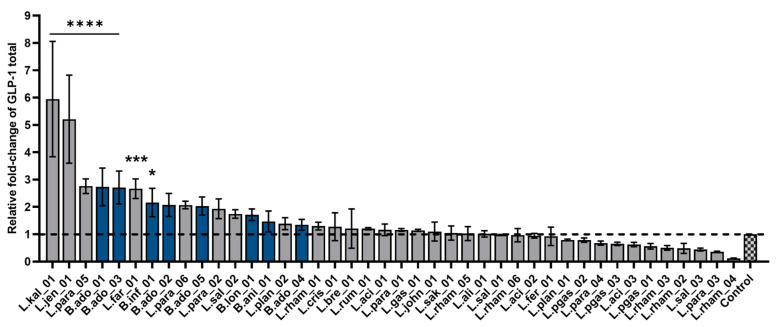
Glucagon-like peptide-1 (GLP-1) secretion from STC-1 cells after bacterial co-incubation. Ranking of 42 bacterial strains based on ability to elicit secretion of GLP-1 from STC-1 monolayers. GLP-1 total was measured after 3 h incubation via the MSD platform. Each blue bar corresponds to *Bifidobacterium* strains and dark gray to *Lactobacillus* strains. Control is untreated cells in HEPES buffer. The black dotted line indicates baseline of control GLP-1 secretion = 1. Data shown as mean of triplicates normalized to baseline and error bars are SD. Results were compared with unstimulated control via a one-way ANOVA with Dunnett’s post-hoc analysis for multiple comparisons: **** *p* < 0.0001, *** *p* < 0.001, * *p* < 0.05 to control test.

To screen for bacterial activation of beneficial gut hormone secretion, in this case, the incretin GLP-1, monolayers from the intestinal secretin tumor cell line STC-1, were used. The enteroendocrine cell model was exposed to live bacteria suspensions (10^9^ CFU/mL) and GLP-1 concentration was measured in the supernatant after 3 h. The 42 strains were ranked based on their GLP-1 secretion potential (Figure 4). Seven strains showed statistically significantly induced secretion of GLP-1 compared with baseline, of which three belonged to the *Bifidobacterium* genus. However, B.ani_01 (1.46-fold increase) and B.ado_04 (1.34-fold increase) did not induce GLP-1 significantly compared with the control. In addition, L.rham_01 did not significantly promote secretion of GLP-1 (1.3-fold increase) compared with unstimulated cells (=1). Interestingly, two bacterial strains, previously not recognized for probiotic abilities, namely L.kal_01 and L.jen_01, dramatically boosted GLP-1 release 5-6-fold compared with the baseline.

### 3.5. Transcriptional Changes in Human Small Intestinal Organoids upon Co-Incubation with Bacteria

To screen the bacterial strains with respect to impacting the small intestine epithelial cells, gene expression of bacterial-stimulated human intestinal derived organoids (HIO) was analyzed. By differentiating 3D HIO into 2D monolayers, bacterial exposure onto the apical side of the polarized intestinal epithelial cells was enabled. Small intestinal-specific cell types were identified by mRNA expression of cell-type specific markers evaluated by transcriptomics analysis (Supplementary Table S3). Here, the following markers indicated the presence of various cell types such as mature enterocytes (via expression of EPCAM, Keratin 20, E-Cadherin 1 and Villin 1), mucus-producing goblet cells (via expression of MUC1, MUC13 and Trefoil factor 3) and Paneth cells (via expression of Lysozyme, CD24 and MMP7).

Six bacterial strains were selected from the list of forty-two (Supplementary Table S1) to be co-incubated with 2D HIO monolayers based on their ability to enhance barrier integrity in Caco-2 monolayers and elicit GLP-1 release from STC-1 cells. The six selected strains were: L.rham_01, L.jen_01, B.lon_01, B.ado_03, L.para_01 and L.kal_01.

**Figure 5:**
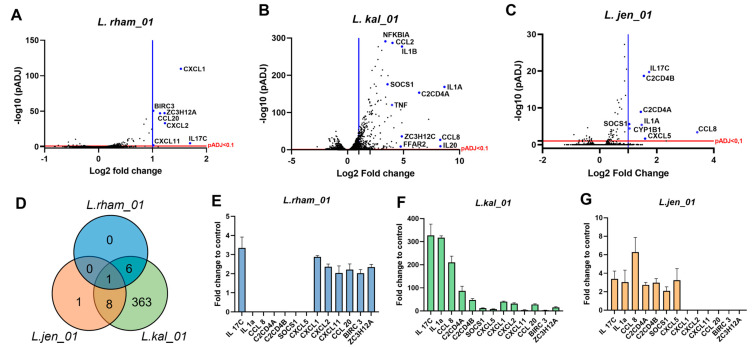
RNA-seq analysis of duodenum-derived organoids upon stimulation with *Lactobacillus* strains. (**A**–**C**) Volcano plot of *Lactobacillus* strains L.rham_01, L.kal_01 and L.jen_01 versus control displaying gene expression by *padj* < 0.1 (red line) correlated to fold change. Right-sided blue line indicates significantly expressed genes (DEG) by log2 fold change >1. (**D**) Venn diagram illustrates common DEG of the *lactobacillus* strains, and the number of genes in common among species. (**E**–**G**) Expression of the common differentially expressed genes (13) identified by Venn diagram. Data are shown as mean of *n* = 3 with error bars indicating SD.

After 2 h of co-incubation, RNA isolation and de novo RNA sequencing, a total of 16,830 genes were identified by DESeq2 analysis on average per sample. Compared with the non-stimulated control, cells stimulated with L.kal_01 had by far the most differently expressed genes (443; DEG), followed by L.jen_01 (9) and L.rham_01 (7). Conversely, B.lon_01- and B.ado_03-stimulated organoid 2D monolayers only had one DEG each, and L.para_01 did not have any DEGs in this setup. Strain-specific volcano plots enable the visualization of total transcriptional changes between control and bacteria-stimulated conditions (Figure 5A–C).

One gene, IL17C, encoding the cytokine interleukin-17C, was significantly upregulated by all three *Lactobacillus* treatments: L.kal_01 with 327-fold, and L.jen_01 and L.rham_01 with 3.4-fold upregulation (Figure 5D). IL17C is known to be produced by intestinal epithelia and rapidly secreted upon specific bacterial stimuli, thus regulating the innate immune response and promoting host defense [59]. IL1A was upregulated by L.kal_01 (537-fold) and L.jen_01 (3.0-fold)-treated HIO monolayers. IL1A is found intracellularly in most epithelial cells and has been shown to be released upon cell necrosis, thus functioning as an alarmin and initiating proinflammatory signaling cascades [60], regeneration and tissue repair [61].

Both L. kal_01 and L.rham_01 stimulation significantly upregulated a repertoire of chemokines: CXCL1, CXCL2 and CCL20, all known for their anti-microbial beneficial defensin-like functions [62]. Another upregulated chemokine exhibiting bactericidal actions, CCL8, was additionally upregulated by both L.kal_01 and L.jen_01.

Another interesting group of upregulated genes comprises NFKBIA, BIRC3 (a ubiquitin-protein ligase), SOCS1 (a suppressor of cytokine signaling), and ZC3H12A (an RNase known to cleave mRNAs encoding IL6 and IL12p40). Together, the products of these genes have shown to downregulate the key inflammation response factor NF-κβ, otherwise rapidly activated upon bacterial stimulation [63,64].

Intriguingly, a subset of free fatty acid receptors, FFARs, was regulated upon treatment with L.kal_01. The short chain fatty acid receptor FFAR2 was upregulated by 4.7-fold (Figure 5B), whereas the long chain fatty acid receptor FFAR4 was downregulated (−0.4-fold). The list of gene ontology (GO) terms generated with the results depicts biologically relevant pathways associated with the significantly regulated genes. Except for L. para_01, the analysis highlights that the *Lactobacillus* strains highly influence pathways for “Immune response” (GO:0006954), “Cellular response to interleukin-1” (GO:0071347) and “Chemokine-mediated signaling” (GO:0070098) (Supplementary Figure S3).

### 3.6. Lipogenesis Inhibition in Primary Rodent Hepatocytes by Bacterial Key Metabolites

**Figure 6:**
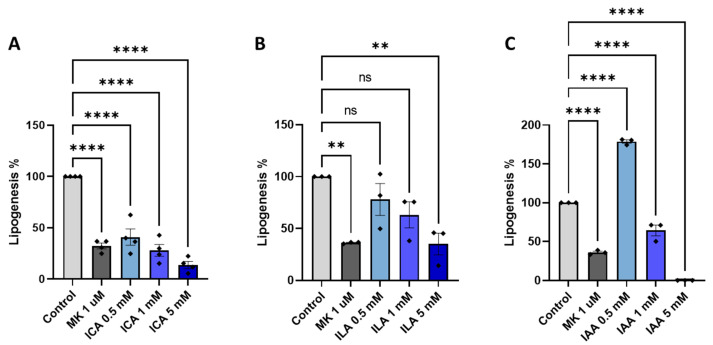
Indole metabolites inhibits de novo lipogenesis in primary hepatocytes. Results of de novo lipogenesis upon 3 h stimulation with high-quantity microbial-derived indole metabolites; indole-3-carbaldehyde (ICA) (**A**), indole-3-carbaldehyde (ILA) (**B**), or indoleacetaldehyde (IAA) (**C**). Allosteric AMPK activator (MK-8722: MK) was used as the positive control. Results are depicted as percentage de novo lipogenesis where media control (vehicle) is set to 100%. Data shown as mean of triplicates (*n* = 3) and error bars are SD. One-way ANOVA followed by Dunnett’s post-hoc analysis for multiple comparisons was performed. **** *p* < 0.0001, ** *p* < 0.01, vs. control.

Once microbial metabolites are absorbed through the intestinal lining, they soon reach the liver via the hepatic portal vein. To determine if any relevant bacterial-derived metabolites had an impact on hepatic lipogenesis, we selected high-quantity produced metabolites from the six strains in the screening pipeline (L.rham_01, L.jen_01, B.lon_01, B.ado_03, L.para_01, and L.kal_01) (Supplementary Table S4). Mouse primary hepatocytes were treated with different doses of selected indole derivatives for 3 h and the inhibition of de novo lipogenesis was assessed and shown as a percentage where vehicle-treatment control was set to 100% (Figure 6). As anticipated, the allosteric AMPK activator MK-8722 (1 μM) robustly inhibited lipogenesis on average by 64% (Figure 6A, B and C). Treatment with indole-3-carbaldehyde (ICA) or indole-3-lactate (ILA) both resulted in a significant and dose-dependent reduction in lipogenesis, with approximately 70% inhibition achieved by ICA at 1 mM, and ILA at 5 mM, and 85% reduction with ICA at 5 mM. On the contrary, indoleacetaldehyde (IAA) (Figure 6C) treatment at low dose resulted in an almost 2-fold activation of de novo lipogenesis, and high doses of IAA resulted in inhibition below the detection limit indicating cytotoxicity, however this was not tested.

## 4. Discussion

The interplay between host and commensal bacteria in health and disease has been an intensively discussed topic in recent years, and the diverse interactions underpin the complexity whereby the gut microbiota and its metabolites influence host homeostasis. The use of microbes as potential therapeutics for management of liver diseases is understudied and poorly understood [65]. Therefore, in this paper, we sought to screen and characterize both well-established probiotic strains as well as more novel, non-characterized bacterial strains as protective agents against the development of NAFLD.

### 4.1. Bacteria and Derived Metabolites’ Effect on Barrier Integrity

First, 42 bacterial strains were assessed in vitro on their ability to increase intestinal barrier tightness due to the fact that “Leaky gut syndrome” defined by increased gut permeability has been reported in NAFLD patients [66,67,68]. In general, *Lactobacillus* species have been widely acknowledged to improve the intestinal barrier and tight junction integrity and, thereby, they could potentially provide protection against a leaky gut epithelial barrier [58]. In concordance with this, we found that the majority of the bacteria included in this screening significantly increased TEER in vitro compared with media control, likely caused by upregulation and/or translocation of tight junction (TJ) proteins such as zonula occludens (ZO-1 and ZO-2), occludin, and claudin, serving as essential inter-cell connectors in epithelial cells [69]. In 2014, Orlando and colleagues demonstrated increased mRNA and protein levels of ZO-1, occludin and claudin-1 in Caco-2 cells treated with viable bacteria from the strain named *Lactocaseibacillus rhamnosus,* L.GG [70]. Additionally, members of other *Lactobacillus* species *(L. rhamnosus, L. plantarum* and *L. casei*) have shown preventive effects by dampening the disruption of TJ proteins and ameliorated barrier function in Caco-2 cells challenged with either LPS or proinflammatory cytokines [71,72].

The TEER responses observed could be influenced by a broad range of bacterial-produced metabolites, including the produced acids. Most lactobacilli grow well under aerobic conditions, whereas bifidobacterium are classified as anaerobic bacteria and are indigenous inhabitants of the distal colon with limited oxygen availability [73,74]. This might have compromised their bacterial fitness and, thus, their metabolite output, resulting in a lower TEER response when compared with the robust survival and metabolic capacities of lactobacilli as facultative anaerobes [74,75]. One example of this is B.ani_01, whose metabolic profile according to our PCA plot is similar to the controls without bacteria, indicating that the strain was metabolically inactive during the TEER assay. Furthermore, the metabolomic profiles from most of the highest-ranking lactobacilli from the TEER assay, clustered together in the PCA plot, indicating that the increase in TEER correlated with the type of produced metabolites.

Interestingly, B.lon_01 induced the highest TEER of all and revealed a unique metabolite profile in the PCA plot. This could partly be explained by the production of acetate, the most abundant SCFA in the bloodstream and colon, and which is a by-product of most anaerobic bacteria in the gut [76]. In fact, B.lon_01 produced the highest concentrations of acetate amongst the strains analyzed, highlighting a key role for this SCFA in reinforcement of intestinal barrier function [77,78,79]. The effect of acetate was previously investigated by Hsieh C.Y. and colleagues, who showed that acetate restored barrier integrity and increased mRNA expression of occludin in a TNFα-challenged Caco-2 monolayer [80].

Another distinct feature of B.lon_01 is the production of multiple indole-metabolites. As for acetate, beneficial effects of indoles have been extensively explored in the gastrointestinal tract and pivotal mechanisms have been characterized for the maintenance of health [32,81,82]. In fact, indoles have been identified as important regulators of not only systemic homeostasis in the host, but also for amelioration of intestinal integrity, hepatic steatosis, and inflammation [41,83,84,85]. In our study, two main indoles; indole-3-lactic acid and indole-3-acetaldehyde were identified in bacterial supernatants, notably that of B.lon_01 (Supplementary Table S3). A study showed that indole (1 mM) prevented epithelial permeability by increasing barrier function and upregulating genes responsible for tight junction and cytoskeleton assembly [84]. Furthermore, it has been hypothesized that indoles inhibit inflammatory pathways in the liver, such as the nuclear factor-κβB (NF- κβ) pathway induced by hepatic Kupffer cells [24]. In addition, an indirect suppression of the NF-κβ pathway was suggested by Zhao and colleagues [41] by indole-propionic acid-induced upregulation of intestinal TJs and subsequent increased intestinal integrity, which prevented passage of endotoxins into the liver, resulting in alleviated liver inflammation in rats. Moreover, using high fat diet induced NAFLD mice models it has been shown that supplementation with indole derivatives (indole, IPA and IAA) protected against fatty liver and downregulated specific lipogenic genes, such as Acetyl CoA carboxylase (ACC), and fatty acid synthase (FAS) [28,41,83,85]. Therefore, based on the increased TEER results and high production of indoles, such as ILA, B.lon_01 was considered a potential candidate for promoting both gut and liver health.

Additionally, microbial-derived metabolites highly associated with a strong TEER response were identified via random forest prediction analysis on the metabolome of the apical supernatant from the bacterial-Caco-2 co-incubation. Choline, hydroxyisocaproic acid, eicosapentanoic acid and indole-3-lactic acid were selected for testing in TEER in concentrations covering the range found in the supernatant. None of the compounds, however, induced a significant TEER response in any of the concentrations tested, suggesting that the TEER responses observed during bacterial co-incubation depend on a combined effect of an array of the secreted metabolites, or that, alongside the bacterial metabolite, a physical contact between bacteria surface structures and cell surface receptors is essential. As an example of this, two soluble proteins (p40 and p75) have been identified in the supernatants of *L. paracasei* and *L. rhamnosus* strains [86]. These proteins are cell wall hydrolases, and p40, especially, has been associated with significant epithelial cell responses by activation of the epidermal growth factor receptor, which subsequently decreases intestinal permeability via the impact on tight and adherents junctions [87]. This could explain the strong and comparable TEER increases for the *L. paracasei* and *L. rhamnosus* strains observed in this study. However, these proteins were not found in *L. acidophilus* supernatants [88], which is in line with our findings where *L. acidophilus* strains were ranked below L.rham_01 (LGG^®^, *Lactocaseibacillus rhamnosus*). It is known that, particularly the *L. rhamnosus* species’ soluble protein, p40, has a fortifying effect on the intestinal epithelium [88,89] and in our conducted TEER screening, the remaining *L. rhamnosus* strains were ranked lower, suggesting intra-species variation. Future proteomic and metabolomic analyses could be set up to reveal the variations between these species.

In general, no clear strain differences were revealed in relation to the metabolome profiles of the screened bacteria, but clear species differences were observed. For example, *L. paracasei* and *L. rhamnosus* species produced formic acid whereas *L. paragasseri* and *L. salivarius* species did not. The obtained knowledge and future analysis of how novel and established probiotic strains cluster with relation to their metabolic profiles, could enable easier and more qualified selection of therapeutic strains for specific target areas. It could further support the investigation into the physiology of mode of action.

### 4.2. Bacterial Effect on GLP-1 Secretion

As previously mentioned, increased GLP-1 levels in circulation have proven to be beneficial for the improvement of metabolic-related diseases, including NASH [37]. The results from the in vitro GLP-1 release screening displayed a more varied effect of the bacterial strains, where seven strains increased GLP-1 release significantly while the remaining strains had no apparent effect on the hormone secretion. The seven secretory stimulating strains were both from the *Lactobacillus* and *Bifidobacterium* species and their respective metabolome profiles are represented in three out of the four PCA plot groups, indicating no simple correlation with the metabolite profiles. However, it is important to note that the metabolic profiles were based on 18 h incubation with Caco-2 cells, whereas the GLP-1 secretion assay only incubated for 3 h.

Despite that, many of the assessed bifidobacteria significantly induced GLP-1 and, as previously mentioned, produced high amounts of acetate. Microbial-derived SCFAs such as acetate not only function as an energy source, positively affecting barrier function, but also as ligands for G-protein-coupled-receptors (GPCRs), known as free fatty acid receptors (FFARs) [23,25]. The major FFARs activated by SCFAs are FFAR2 and FFAR3 [23,90,91], and upon activation of these receptors, pathways involved in glucose and lipid metabolism are regulated [92,93]. In fact, enteroendocrine GLP-1-producing cells have been found to exhibit high expression levels of functional FFAR2 and FFAR3 receptors, leading to secretion of gut hormones such as GLP-1 [30]. In addition, indoles can modulate the secretion of gut hormones from EECs [31] and in this dataset we found overall higher production of indole-3-carbaldehyde secreted from bifidobacteria compared with lactobacilli, which additionally could explain our GLP-1 secretory results, indicating why the *Bifidobacterium* genus overall tends to be a better inducer of GLP-1 than the *Lactobacillus* genus.

Two novel strains, L.kal_01 and L.jen_01, exerted the strongest GLP-1 release under the tested conditions. Little is known about these strains and the absence of substantial evidence on their phenotype limits the mode of action understanding of GLP-1 release and barrier integrity. However, our metabolomic analysis showed that these two strains cluster in the same PCA plot group, suggesting that they, to some extent, display similar metabolite profiles. Specifically, both L.kal_01 and L.jen_01 produce significant levels of 3-methyladenine, a well-known intracellular inhibitor of autophagy [94], and 3-methylxanthine, which is a member of the methylxanthine family known for raising intracellular cAMP levels and thereby promoting increased secretory responses [95]. Future research addressing the effect of these specific metabolites on GLP-1 secretion will provide more answers.

### 4.3. Bacterial Effect on Gene Expression of Human Intestinal Organoids

During the last decade, intestinal organoids have been widely applied as gastrointestinal pre-clinical models where the multilineage morphology of the gut is maintained, allowing researchers to study human intestinal physiology and response to stimuli and challenges in vitro [96,97,98]. Here, we integrated a cutting-edge co-culture technique that aimed to mirror the bacteria-host communication in the gut. Transcriptomic analysis on our bacteria-2D organoid co-incubation showed extremely varying degrees of transcriptional impact with the different bacterial species employed.

The interleukin, IL17C, was the only identified commonly regulated gene for L.kal_01, L.jen_01, and L.rham_01. The significant upregulation of IL17C was consistent with previous findings showing that bacteria and inflammatory challenges can increase IL17C secretion from epithelial tissue, which in an autocrine manner induces expression of proinflammatory chemokines and cytokines [59]. A possible activation pathway of IL17C secretion could be via stimulation of toll-like receptors TLR2 and TLR5, that recognize bacterial lipoprotein and flagellin, respectively, as described by Ramirez-Carrozzi et al. Interestingly, the authors discovered that IL17C played an important role in maintaining intestinal epithelial homeostasis after an inflammatory challenge by showing how colonic intestinal damage and disease severity were significantly lower in wild type DSS-treated mice compared with IL17C knock-out DSS-treated mice.

Another interesting observation was the common upregulation of the chemokines CXCL1, CXCL2, CCL8 and CCL20, which were proven to display anti-microbial defensin-like activities [62]. The chemokines all displayed varying degrees of killing or inhibiting growth of pathogenic bacteria, such as *S. pyogenes, M. catarrhalis, P. aeruginosa, E. coli* and *S. aureus* [62]. By inducing chemokines that inhibit pathogens in the small intestine, NAFLD-associated dysbiosis such as SIBO and increased LPS translocation could be ameliorated or prevented. These genes were also shown to be upregulated in a study by O’Callaghan and colleagues (2012) where they stimulated Caco-2 cells with *Ligilactobacillus salivarius* (previously known as *Lactobacillus salivarius)* and observed similar antimicrobial properties [64]. Furthermore, a group of genes, namely, NFKBIA, BIRC3, SOCS1 and ZC3H12A, attributed to dampening or inhibiting an acute proinflammatory response via NF- κβ signaling, were also upregulated with L.rham_01, L.kal_01, and L.jen_01 co-incubation. This combination of genes remarkably also appeared upregulated in Caco-2 cells stimulated with *L. salivarius*, such as the chemokines mentioned above, indicating a common *Lactobacillus* inflammatory regulatory response. We hypothesize that despite an acute activation of pro-inflammatory pathways upon bacterial co-incubation, the upregulation of the above-mentioned proteins will provide a negative feedback modulating and regulating the NF-κβ response, thus, serving as a compensatory mechanism for surveillance and alertness against opportunistic pathogens, resulting in a mutualistic relationship between microbe and host.

### 4.4. Bacterial-Derived Metabolites’ Effect on Hepatic Lipogenesis

In addition to improved barrier integrity and stimulation of GLP-1 secretion, another attributable feature of indole metabolites is hepatoprotective effects [81,99,100,101]. Ma L. and colleagues (2020) reported that indole levels were reduced in the plasma of obese humans and diet-induced NAFLD mice, and oral administration with indole (50 mg/kg) for four weeks ameliorated liver steatosis in mice. Intriguingly, this protective effect of fatty liver with indole treatment was associated with reduced hepatic mRNA levels of proinflammatory and lipogenic genes (e.g., *FAS* and *ACC*) [85]. With the semi-polar analysis conducted in this study, indole was not measured, but has been reported to act via stimulation of AhR in a similar fashion to most indole derivates [102]. We observed a dose-dependent inhibitory effect of ICA and ILA on hepatic de novo lipogenesis in murine primary hepatocytes, which hypothetically could explain the protective effect of orally delivered indole and ICA found on fatty liver in vivo in multiple studies [85,103,104]. Thus, indole and indole-derivate high-producing bacterial strains are speculated to be promising candidates for probiotics promoting liver health.

## 5. Conclusions

In this study, we aimed to identify candidate bacterial strains with prophylactic properties against NAFLD. We set up a liver health-relevant in vitro assay pipeline to screen the ability of a set of viable bacteria to strengthen the gut barrier, boost GLP-1 secretion, affect organoid transcriptomic profile, and inhibit hepatic lipogenesis in various in vitro assays to identify potential liver-health-promoting bacteria. These results suggest that viable bacteria and/or collective microbial-derived metabolites are needed for the improvement of the integrity of the gut lining. Via TEER results and metabolome profiling, the B.lon_01 strain was established as a possible candidate as it induced the highest increase in TEER and had a unique position in the PCA plot due to its production of indole-3-lactic acid and indole-3-acetaldehyde, which are metabolites with recognized beneficial physiological actions, including, as shown herein, inhibition of hepatic lipogenesis. Furthermore, L.kal_01 and L.jen_01 are two promising strains that revealed positive findings in the different in vitro setups. The results from this screening study may contribute to future studies investigating the translatability of the beneficial traits in multi-organ, whole-body in vivo or clinical setups. Further investigation of bacterial phenotypical traits such as bacteria competition assays and multi-strain analysis are strongly suggested for the development of next-generation probiotics as prophylactic therapy for NAFLD. High-quality and physiologically relevant pre-clinical screenings build a solid foundation for choosing better and more beneficial probiotic strains, thus increasing the possibility of success when moving into clinical studies.

## Supplementary Materials

The following supporting information can be downloaded at: https://www.mdpi.com/article/10.3390/nu15102361/s1. Figure S1: Flow chart of the in vitro screening pipeline. To enable the finding of possible candidate probiotic strains for improved metabolic health such as liver health, a library of 42 strains from *Lactobacillus* and *Bifidobacterium* genus were screened. Barrier integrity were evaluated by measuring TEER in Caco-2 cells and activation of GLP-1 secretion were measured from STC-1 cell line. Microbial-derived metabolites from spent media were profiled from all 42 strains and key metabolites were identified. Key metabolites were tested for beneficial effects in primary murine hepatocytes and with relation to TEER with Caco-2 cells. The six strains that collectively performed the best in TEER and GLP-1 secretion assay were further screened in relation to intestinal epithelial transcriptional changes using 2D human small intestinal-derived organoids. Figure S2: Transepithelial electrical resistance (TEER) assay upon stimulation with bacteria. A–G) TEER normalized to baseline t = 0 set as 100% barrier integrity with 8 hrs. in co-culture with viable bac-teria. DMEM = AB-free as the negative control and L.rham_01 as positive control. Co-cultures were tested in triplicates (n = 3) and error bars indicate SD. Figure S3: Gene ontology analysis (GO) Lactobacilli strains. A) L.rham_01, B) L.kal_01 and C) L.jen_01. Table S1: Bacteria strain library. LGG^®^, GR-1^®^, LA-5^®^, LA-2^®^, L.CASEI 01TM, L.CASEI 431^®^, ISTILOSTM and BB-12TM are trademarks of Chr. Hansen A/S. Table S2: Metabolites tested in TEER. Table S3: 2D human intestinal organoid monolayer cell types. Values represent mean Transcripts Per Million (TPM) from the DESeq analysis (n = 3). Table S4: Highly produced metabolites. Values represent the log2 fold change value to the media control without bacteria. (n = 3)
