# Supplementary material for: In Vitro Screening for Probiotic Properties of Lactobacillus and Bifidobacterium Strains in Assays Relevant for Non-Alcoholic Fatty Liver Disease Prevention
Source: Nutrients. 2023 May 18;15(10):2361. doi: 10.3390/nu15102361 (PMC10224198; doi:10.3390/nu15102361)
Supplement: Supplementary file 1 [file nutrients-15-02361-s001.zip › nutrients-2384076-supplementary.pdf]

Supplementary figures and tables

Sup. Figure S1: **Flow chart of the *in vitro* screening pipeline.**

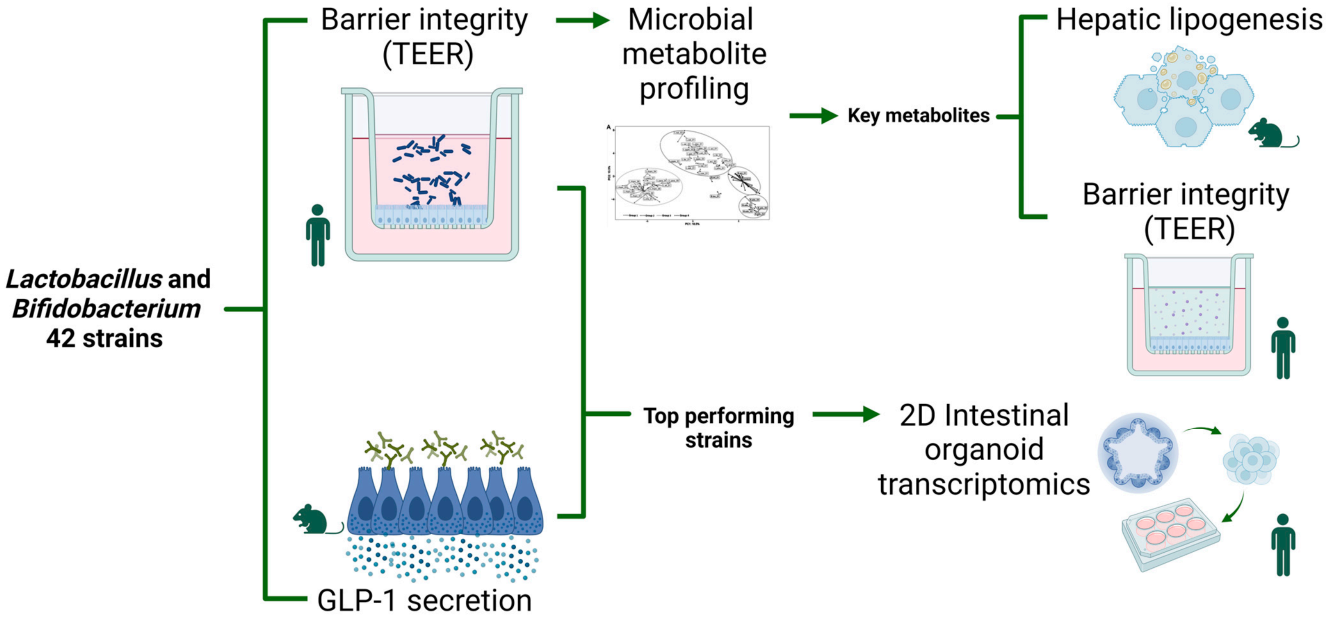

Sup. Figure S1: Flow chart of the *in vitro* screening pipeline. To enable the finding of possible candidate probiotic strains for improved metabolic health such as liver health, a library of 42 strains from *Lactobacillus* and *Bifidobacterium* genus were screened. Barrier integrity were evaluated by measuring TEER in Caco-2 cells and activation of GLP-1 secretion were measured from STC-1 cell line. Microbial-derived metabolites from spent media were profiled from all 42 strains and key metabolites were identified. Key metabolites were tested for beneficial effects in primary murine hepatocytes and with relation to TEER with Caco-2 cells. The six strains that collectively performed the best in TEER and GLP-1 secretion assay was further screened in relation to intestinal epithelial transcriptional changes using 2D human small intestinal-derived organoids.

Sup. Table S1: Bacteria strain library

Strain(Genus, Species, Subspecies)			DSM number	Trademark	ID Code
Lactocaseibacillus	*rhamnosus*		DSM33156	LGG®	L.rham_01
Lactocaseibacillus	*rhamnosus*			NA	L.rham_02
Lactocaseibacillus	*rhamnosus*			NA	L.rham_03
Lactocaseibacillus	*rhamnosus*			NA	L.rham_04
Lactocaseibacillus	*rhamnosus*			NA	L.rham_05
Lactobacillus	*rhamnosus*		DSM33426	GR-1®	L.rham_06
Lactobacillus	*paragasseri*			NA	L.pgas_01
Lactobacillus	*paragasseri*			NA	L.pgas_02
Lactobacillus	*paragasseri*			NA	L.pgas_03
Lactobacillus	*gasseri*			NA	L.gas_01
Lactobacillus	*acidophilus*		DSM13241	LA-5®	L.aci_01
Lactobacillus	*acidophilus*			NA	L.aci_02
Lactobacillus	*acidophilus*		DSM32754	LA-2®	L.aci_03
Lacticaseibacillus	*paracasei*			NA	L.para_01
Lacticaseibacillus	*paracasei*			NA	L.para_02
Lacticaseibacillus	*paracasei*	*paracasei*	DSM19465	L.CASEI 01™	L.para_03
Lacticaseibacillus	*paracasei*			NA	L.para_04
Lacticaseibacillus	*paracasei*	*paracasei*	DSM33451	L. CASEI 431®	L.para_05
Lacticaseibacillus	*paracasei*			NA	L.para_06
Ligilactobacillus	*salivarius*			NA	L.sal_01
Ligilactobacillus	*salivarius*			NA	L.sal_02
Ligilactobacillus	*salivarius*	*salivarius*	DSM33820	NA	L.sal_03
Lactobacillus	*crispatus*			NA	L.cris_01
Companilactobacillus	*alimentarius*			NA	L.ali_01
Lactobacillus	*jensenii*			NA	L.jen_01
Latilactobacillus	*sakei*			NA	L.sak_01
Companilactobacillus	*farciminis*			NA	L.far_01
Lactobacillus	*plantarum*			NA	L.plan_01
Lactobacillus	*plantarum*			NA	L.plan_02
Ligilactobacillus	*ruminis*			NA	L.rum_01
Lactobacillus	*kalixensis*			NA	L.kal_01
Levilactobacillus	*brevis*			NA	L.bre_01
Lactobacillus	*johnsonii*			NA	L.john_01
Limosilactobacillus	*fermentum*			NA	L.fer_01
Bifidobacterium	*longum*	*longum*	DSM15955	NA	B.lon_01
Bifidobacterium	*adolescentis*			NA	B.ado_01
Bifidobacterium	*adolescentis*			NA	B.ado_02
Bifidobacterium	*adolescentis*			NA	B.ado_03
Bifidobacterium	*adolescentis*			NA	B.ado_04
Bifidobacterium	*adolescentis*			NA	B.ado_05
Bifidobacterium	*longum*	*infantis*	DSM33361	Bifin02, ISTILOS™	B.inf_01
Bifidobacterium	*animalis*	*lactis*	DSM15954	BB-12™	B.ani_01

LGG®, GR-1®, LA-5®, LA-2®, L.CASEI 01™, L.CASEI 431®, ISTILOS™ and BB-12™ are trademarks of Chr. Hansen A/S.

Sup. Table S2: Metabolites tested in TEER

Metabolite	Concentration (μM)	Product ID
DL-Indole-3 lactic acid	4180; 418; 41.8; 4.18	Sigma I5508
Choline chloride	600; 60; 6	Sigma C7017
(S)-(-)-2-Hydroxyisocaproic acid (HI-A)	3930; 393; 39.3	Sigma 219827
cis-5,8,11,14,17-Eicosapentaenoic acid	20; 2; 0.2	Sigma 44864

Sup. Figure S2: Transepithelial electrical resistance (TEER) assay upon stimulation with bacteria.

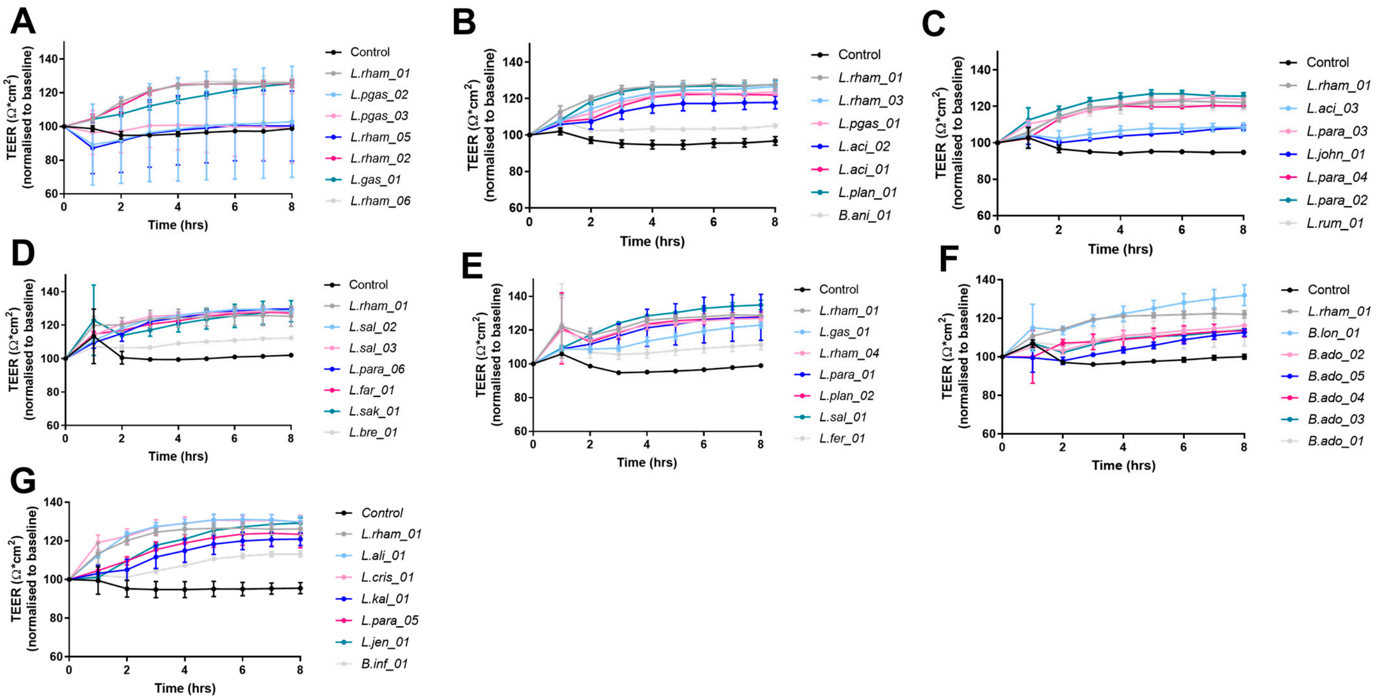

Sup. Figure S2) Transepithelial electrical resistance (TEER) assay upon stimulation with bacteria. A-G) TEER normalized to baseline t=0 set as 100% barrier integrity with 8 hrs. in co-culture with viable bacteria. DMEM = AB-free as the negative control and L.rham_01 as positive control. Co-cultures were tested in triplicates (n=3) and error bars indicate SD.

Sup. Table S3: 2D human intestinal organoid monolayer cell types

Intestinal cell types in 2D monolayer cultures						
**Cell type**	**Gene name**		**Control A**	**SD**	**Control B**	**SD**
**Enterocyte**	EPCAM	Epithelial cell adhesion molecule	4288,4	61,8	3262,5	85,4
	ALPI	Alkaline Phosphatase	149,3	1,7	55,3	7,7
	CDH1	E-cadherin 1	473,1	23,8	401,7	66,0
	KRT20	Keratin 20	2118,0	85,2	1567,2	120,2
**Stem cell/proliferation**	MKI67	Marker Of Proliferation Ki-67	5,2	0,2	5,6	1,2
	LGR5	Leucine Rich Repeat Containing G Protein-Coupled Receptor 5	0,6	0,1	0,6	0,0
	BMI1	BMI1 Proto-Oncogene	11,7	0,4	12,9	0,5
**Goblet cell**	MUC1	Mucin 1	1284,5	7,7	1032,7	82,6
	MUC2	Mucin 2	2,2	0,2	2,6	0,6
	MUC5B	Mucin 5B	11,2	1,7	2,9	0,8
	MUC13	Mucin 13	5538,3	112,0	3271,5	292,9
	TFF3	Trefoil factor 3	2445,5	49,2	2711,2	69,7
**Paneth cell**	LYZ	Lysozyme	3272,9	70,9	2753,6	151,2
	CD24	CD24 Molecule	948,3	42,0	951,6	74,5
	MMP7	Matrix Metallopeptidase 7	501,5	27,2	1039,2	40,6
**Enteroendocrine cell**	CHGA	Chromgranin A	0,2	0,0	0,1	0,0
	CCK	Cholecystekinin	0,7	0,1	2,6	0,5
	SCT	Secretin	2,6	0,8	0,9	0,1
	REG4	Regenerating islet-derived protein 4	7,8	0,9	56,1	4,4
	NTS	Neurotensin	4,3	0,1	2,5	0,5
**Crypt marker**	CD44	CD44 Molecule	18,8	0,7	34,5	1,4
**Villus marker**	VIL1	Villin1	314,3	12,0	221,2	20,3
**Tight junctions**	TJP1	Zonula occludens 1	51,2	1,1	50,4	6,8
	TJP2	Zonula occludens 2	59,5	1,3	49,3	3,0
	CLDN3	Claudin 3	1292,4	56,6	1187,7	26,2
	CLDN4	Claudin 4	1034,6	15,9	1117,6	72,6
	CLDN7	Claudin 7	1588,7	59,2	1229,2	91,0
	OCLN	Occludin	63,8	3,1	48,5	7,1
**Transmembrane sensors**	FFAR4	Free fatty acid receptor 4	4,5	0,2	10,0	0,5
	TLR1	Toll-like receptor 1	2,0	0,1	1,5	0,1
	TLR2	Toll-like receptor 2	2,5	0,1	1,8	0,0
	TLR3	Toll-like receptor 3	24,6	1,1	15,1	3,0
	GPRC5A	G protein-coupled receptor 5A	640,0	12,7	874,6	61,7
	GPR35	G protein-coupled receptor 35	72,0	2,5	49,6	2,2
**Immune response**	CXCL1	Interleukin-1	68,6	3,3	207,1	2,7
	CXCL3	Interleukin-3	23,3	2,1	86,7	5,0
	CXCL8	Interleukin-8	35,7	2,4	188,3	8,3

Values represent mean Transcripts Per Million (TPM) from the DESeq analysis (n=3).

Sup. Figure S3: Gene ontology analysis (GO) Lactobacilli strains.

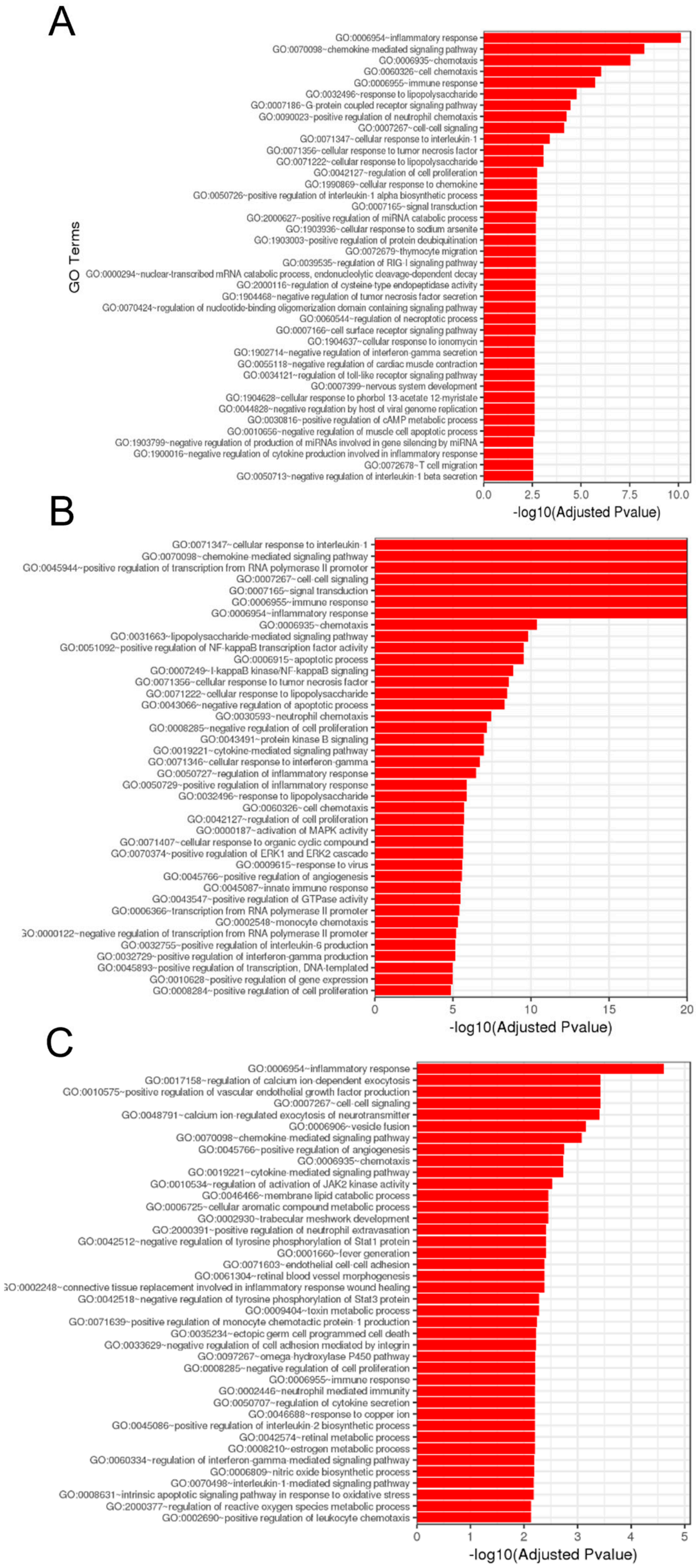

Sup. Figure S3. Gene ontology analysis (GO) Lactobacilli strains. A) L.rham_01, B) L.kal_01 and C) L.jen_01.

Sup. Table S4. Highly produced metabolites

	Metabolites	L.rham_01	L.para_01	L.kal_01	L.jen_01	B.lon_01	B.ado_03
1	2-(methylsulfonyl)-1,2,3,4-tetrahydroisoquinoline	35,24	1,20	3,95	11,00	26,91	0,78
2	2,3,4,5-tetrahydrodipicolinic acid	6,43	1,61	3,00	10,74	2,55	13,81
3	2-aminobutyric acid	0,95	1,13	0,88	1,15	0,91	8,81
4	3-Methyladenine	32,50	39,00	780,13	773,13	1,79	5,40
5	3-Methylxanthine	1,64	2,50	5,04	5,57	1,04	2,29
6	3-Phenyllactic acid	76,94	91,66	187,92	1,32	16,80	1,07
7	4-Indolecarbaldehyde	1,85	2,61	6,07	1,78	8,80	8,86
8	4-Piperidinecarboxamide	5,08	6,53	1,26	1,25	1,19	1,15
9	4-Vinylphenol	77,17	91,53	187,18	1,30	16,87	1,07
10	6-Methyl[1,2,4]triazolo[4,3-b]pyridazin-8-ol	3,10	12,09	94,82	100,46	0,56	1,72
11	7-Methylguanine	2,74	4,86	13,77	21,51	2,36	2,55
12	Acetylarginine	0,97	0,91	4,82	1,06	7,54	6,68
13	Acetylmuramic acid	6,74	0,31	23,66	19,04	0,39	5,03
14	alpha-Hydroxyisovaleric acid	8,67	11,55	8,24	1,47	0,74	0,80
15	Carbamoylaspartate	8,99	588,87	0,85	1,16	2,05	2,50
16	Cytidine	0,95	1,59	0,53	1,00	1,45	6,22
17	Deoxyinosine	1,32	1,31	5,40	6,14	0,85	1,90
18	DL-4-Hydroxyphenyllactic acid	4,60	10,56	9,04	0,91	3,99	0,88
19	D-α-Hydroxyglutaric acid	5,94	19,77	23,15	1,45	9,93	4,04
20	Furfural	85,61	63,42	6,42	0,79	2,19	0,93
21	Guanine	5,87	0,12	8,53	5,32	1,60	11,53
22	Hexanoylglycine	116,11	12,11	11,86	2,50	3,89	2,19
23	Hydroxyisocaproic acid	197,51	275,82	151,72	30,11	6,84	2,15
24	Indole-3-acetaldehyde	1,87	4,57	5,52	0,91	97,88	1,88
25	Indole-3-lactic acid	1,93	4,67	4,90	1,00	62,69	1,15
26	Inosine	10,77	1,67	3,98	0,58	3,12	0,85
27	Isovaleraldehyde	185,59	256,67	142,14	28,11	6,67	2,09
28	Methionine sulfoxide	1,22	1,52	2,21	7,16	1,19	1,24
29	N-Acetylglutamine	15,45	2,25	6,37	28,72	5,66	34,35
30	N-acetylglycine	1,57	1,30	1,78	4,52	1,19	1,17
31	N-Acetylmethionine	11,30	7,09	8,12	7,59	14,24	8,69
32	N-Acetyl-phenylalanine	40,76	11,23	4,03	5,20	42,63	19,03
33	N-Acetyltryptophan	8,83	30,15	4,00	6,69	38,03	65,06
34	N-Acetyltyrosine	8,91	5,71	2,33	3,09	5,06	3,84
35	N-formylmethionine	1,90	3,39	8,19	1,86	1,08	1,72
36	Nicotinic acid	408,87	1355,83	866,30	33,08	4,33	4,67
37	Pipecolinic acid	2,23	9,68	1,12	0,88	2,44	1,13
38	Pyridine	15,29	13,82	7,41	1,07	9,13	9,08
39	Sorbic acid	97,25	5,95	3,84	1,50	0,70	0,78
40	Thymine	21,88	38,74	42,99	28,79	1,76	1,70

Values represent the log2 fold change value to the media control without bacteria. (n=3)
